# The oncogenic role of HIF-1α/miR-182-5p/ZFP36L1 signaling pathway in nasopharyngeal carcinoma

**DOI:** 10.1186/s12935-021-02177-3

**Published:** 2021-08-31

**Authors:** Gang Wang, Fangzheng Zhou, Tong Ou, Haiyan Sun, Zhirui Shan, Yingshen Lu, Gui Chen, Simin Yuan, Xiaowen Zhang, Song Wu

**Affiliations:** 1 Following Precision Medical Research Institute, Shenzhen Luohu People’s Hospital, Shenzhen, 518000 People’s Republic of China; 2 Department of Radiation Oncology, The Third Affiliated Hospital of Shenzhen University (Shenzhen Luohu People’s Hospital), Shenzhen, 518000 People’s Republic of China; 3 Medical Laboratory, The Third Affiliated Hospital of Shenzhen University (Shenzhen Luohu People’s Hospital), Shenzhen, 518000 People’s Republic of China; 4 State Key Laboratory of Respiratory Disease, Department of Otolaryngology-Head and Neck Surgery, The First Affiliated Hospital of Guangzhou Medical University, Guangzhou, 510120 People’s Republic of China

**Keywords:** miR-182-5p, ZFP36L1, HIF-1α, Tumorigenesis, Metastasis, Nasopharyngeal carcinoma

## Abstract

**Background:**

Accumulating evidence indicates that dysregulation of miR-182-5p can serve as diagnostic and prognostic biomarkers for some cancers, whereas the role of miR-182-5p has not been explored in nasopharyngeal carcinoma (NPC). Our study aims to elucidate the biological function of miR-182-5p in NPC and the potential molecular mechanism involved.

**Methods:**

Quantitative real-time polymerase chain reaction (qRT-PCR) was performed to determine miR-182-5p expression in NPC primary tissues and cell lines. Immunohistochemistry (IHC) for ZFP36L1 was conducted in NPC samples. Western blot was used to evaluate protein expression in cell lines. A series of functional assays were carried out to evaluate the roles of miR-182-5p and ZFP36L1 in tumor development and progression of NPC. Bioinformatics tools and luciferase reporter assays were utilized to identify the potential mechanisms of action. Moreover, rescue experiments were applied to explore whether ZFP36L1 mediated the effects of miR-182-5p in NPC.

**Results:**

Up-regulation of miR-182-5p was significantly associated with tumor development and poor prognosis in patients with NPC. Functional study demonstrated that miR-182-5p overexpression enhanced, whereas suppression of miR-182-5p impeded NPC cell proliferation, migration, tumorigenesis and metastasis. Mechanistically, miR-182-5p interacted with ZFP36L1 at two sites in its 3′ un-translated region (UTR) and repressed ZFP36L1 expression in NPC. Consistently, an inverse correlation was observed between the expression levels of miR-182-5p and ZFP36L1 using clinical NPC tissues, and down-regulation of ZFP36L1 in NPC predicts poor survival. Furthermore, overexpression of miR-182-5p in NPC was partly attributable to the transcriptional activation effect induced by hypoxia-inducible factor 1α (HIF-1α).

**Conclusions:**

Our data suggests that miR-182-5p facilitates cell proliferation and migration in NPC through its ability to down-regulate ZFP36L1 expression, and that the HIF-1α/miR-182-5p/ZFP36L1 axis may serve as a novel therapeutic target in the management of NPC.

**Supplementary Information:**

The online version contains supplementary material available at 10.1186/s12935-021-02177-3.

## Background

Nasopharyngeal carcinoma (NPC) is a highly prevalent malignancy in Southeast Asia, especially in southern China [1]. Despite tremendous progress in diagnosis and multimodality therapies, there are still approximately 30% of NPC patients suffering treatment failure and more than 20% of cases eventually develop distant metastasis after radical treatment [2–4]. Identifying novel and effective therapeutic strategies is essential for further improvement of the survival outcome of patients with NPC. However, the molecular mechanisms of NPC development and progression are poorly understood.

MicroRNAs (miRs) are a family of endogenous, small non-coding, ~ 22 nucleotide RNA molecules that can play pivotal regulatory roles in animals and plants by targeting mRNAs for cleavage or translational repression [5]. Numerous studies have demonstrated that dysregulation of miRs contributes to tumorigenesis and progression in many cancer types including NPC and can be associated with prognosis [6–8]. As a member of the highly conserved miR-183-96-182 cluster [9], miR-182 is located on human chromosome 7q32.2 [10]. Recent data shows that miR-182 may play an oncogenic role in many human cancers, including the breast cancer [11, 12], colorectal cancer [13], prostate cancer [14] and bladder cancer [15]. However, the detailed functions and underlying mechanisms of miR-182-5p in NPC have not been explored.

Here, we validated that miR-182-5p played a crucial role in promoting NPC tumorigenesis and metastasis by down-regulating ZFP36L1. In addition, either miR-182-5p or ZFP36L1 was found to be valuable for the prognosis of outcome in patients with NPC. Moreover, we revealed that HIF-1α appeared to mediate the overexpression of miR-182-5p in NPC.

## Materials and methods

### Clinical samples

Seventy-five primary NPC samples were collected from September 2009 to May 2013 at The First Affiliated Hospital of Guangzhou Medical University. None of the patients had received chemotherapy or radiation therapy before biopsy. The informed consent was obtained from each patient. Clinical characterizations of these patients are summarized in Table 1. This study was approved by the Ethics Committee of The First Affiliated Hospital of Guangzhou Medical University and the Ethics Committee of Shenzhen Luohu People’s Hospital.

**Table 1:** Patients and tumor characteristics

Characteristics	No. of cases (%)
Age, years	
Median	52
Range	26–70
Gender	
Male	57 (76.0)
Female	18 (24.0)
T classification	
T_1_	10 (13.3)
T_2_	16 (21.3)
T_3_	33 (44.0)
T_4_	16 (21.3)
N classification	
N_0_	16 (21.3)
N_1_	29 (38.7)
N_2_	22 (29.3)
N_3_	8 (10.7)
M classification	
M0	75 (100.0)
M1	0 (0.0)
Death	
No	57 (76.0)
Yes	18 (24.0)
Clinical stage	
I	1 (1.3)
II	17 (22.7)
III	34 (45.3)
IV	23 (30.7)

Clinical stage was determined according to 8th edition of UICC-AJCC TNM staging system

### Immunohistochemistry (IHC)

This procedure was performed as previously described [16, 17]. In short, paraffin sections were deparaffinized using xylene and rehydrated using a series of alcohol solutions. Endogenous peroxidase activity was blocked by treatment with 3% H_2_O_2_ for 15 min. Then the sections were incubated with an anti-ZFP36L1 (Abcam, 1:100) antibody overnight at 4 °C. After washing with PBS three times, the tissue slides were treated with secondary antibody using a MaxVision™ HRP-Polymer anti-Rabbit IHC Kit (Maixin, China). The color was developed using a DAB Horseradish Peroxidase Color Development Kit (Maixin, China), and the sections were counterstained with haematoxylin. The degree of immunostaining was scored according to both the proportion of positively stained tumor cells and the staining intensity. The score for each tissue was calculated by multiplying the staining index (0, 1, 2 and 3) by the percentage category value (0, 1, 2, 3 and 4), and the average of the scores from the two pathologists was used as the final score.

### Oligonucleotides and plasmids

The miR-182-5p mimic/shRNA (182-mimic/182-shRNA), the appropriate negative controls (Control-mimic/Control-shRNA) were obtained from Genepharma (Shanghai, China). 182-shRNA-expressing lentivirus was used to stably transfect NPC cells to establish miR-182-5p-silencing cells. The ZFP36L1 siRNA (siZFP36L1, 5′-AACTATAGTGCTCCCAGTGC-3′) and control non-targeting siRNA (siControl) were synthesised by Ribobio (Guangzhou, China). ZFP36L1 and HIF-1α were cloned into the pcDNA3.1 vector. For cell transfection, Lipofecamine 2000® Transfection Reagent (Invitrogen, USA) was used according to the manufacturer’s guidelines.

### Cell culture

Human NPC cell lines (HK1, CNE1) were obtained from the cell bank of the Chinese Academy of Science. These cells were maintained in DMEM medium supplemented with 10% fetal bovine serum (Hyclone, USA), 100 units/ml penicillin and 100 mg/ml streptomycin (Beyotime Biotech, China) and incubated in humidified air at 37 °C under a 5% CO_2_ atmosphere. To induce hypoxia, cells were cultured in a hypoxic incubator (WAKENYAKU CO., Japan) at 1% oxygen tension for 0 h, 24 or 48 h. All cell lines used in this study were regularly authenticated by morphological observation and haven’t been in culture for more than 2 months.

### RNA extraction and qRT-PCR

Total RNA was isolated with TRIzol (Invitrogen, USA) according to the manufacturer’s instructions. cDNA was synthesized with the PrimeScript^RT^ reagent Kit (Promega, USA). qRT-PCR was carried out using the SYBR Green SuperMix kit (Roche, Switzerland) and ABI 7900HT Fast Real-Time PCR system (Applied Biosystems). U6 was used as the internal control. The primers of miR-182-5p and U6 were purchased from Ribobio.

### Western blot

Briefly, cells were lysed with a radioimmunoprecipitation assay (RIPA) lysis buffer (Beyotime Biotech, China). Aliquots (30–50 µg) of proteins were resolved by SDS-PAGE (10%), then electro-transferred onto polyvinylidene difluoride membranes (Merck Millipore, USA) and immunoprobed. The protein-antibody complexes were detected using the ECL detection reagents (Beyotime Biotech, China). Primary antibodies used: anti-ZFP36L1 (ab42473), anti-HIF-1α (ab1) and anti-Hsp70 (ab5439) from Abcam (Cambridge, USA).

### The CCK-8 assay

The cell viability was measured with the CCK-8 kit (Dojindo, Japan) according to the manufacturer’s protocol. Cells at the logarithmic phase were seeded into 96-well plates at the density of 1500 cells/well, and then incubated for 24 h, 48 h, 72 or 96 h after transfection. The optical density (OD) of each well was measured at a wave length of 450 nm.

### Cell migration assay

This assay was performed in a Transwell chamber with 8 μm pores (Corning, USA). 1 × 10^5^ cells in 200 µl serum-free DMEM medium were seeded into the top chamber of wells inserted in a 24-well plate, while the bottom chamber was filled with 600 µl medium containing 20% FBS. After 24 h incubating, cells on the bottom of the membrane were fixed with 4% paraformaldehyde at room temperature for 30 min, stained with 0.1% crystal violet for 15 min and the cell number was counted in five randomly selected fields under an inverted microscope (Nikon, Japan).

### Luciferase reporter assay

A 1306 bp fragment of ZFP36L1 3′ UTR containing the putative miR-182-5p binding sites, the corresponding mutated 3′ UTR of ZFP36L1 and the promoter region (~ 2400 bp) of miR-182-5p were amplified and directly cloned into the pGL3.0 basic vector (Promega, USA). Cells were also co-transfected with the pRL-TK vector (Promega, USA), which served as an internal control to normalize transfection efficiency. Dual luciferase signals were measured 48 h later by the Dual-luciferase assay kit (Promega, USA) following the manufacturer’s protocols.

### Animal experiments

These procedures were performed as described previously [18]. All animal experiments were in accordance with animal welfare and European animal care guidelines, and were approved and supervised by the Ethics Committees. The 4-week-old SPF grade female nude mice were obtained from the SLAC Laboratory Animal Center (Shanghai, China). Mice were euthanized by dislocation of cervical vertebra at the end of the experiment. For tumorigenesis assay, 2 × 10^6^ HK1 cells stably transfected with 182-shRNA or Control-shRNA were subcutaneously injected into the double dorsal flanks of nude mice. Tumors were harvested 4 weeks after injection and individually weighted. For the in vivo metastasis model, 1 × 10^6^ HK1 cells suspended in 100 µl normal saline were injected intravenously through the tail vein. After 6 weeks, the mice were sacrificed and lung metastatic nodules were carefully examined and counted.

### Statistical analysis

Results were expressed as mean ± SD of at least three independent experiments. SPSS standard V.17.0 (SPSS Inc., IL) was used for Statistical analysis. Student’s *t*-test, Mann–Whitney test and Chi-square test were used to analysis the significance of difference. Survival analysis was performed using Kaplan–Meier plots and log-rank tests. Difference was considered to be statistically significant when p < 0.05.

## Results

### Up-regulation of miR-182-5p is associated with NPC development and poor prognosis

**Fig. 1:**
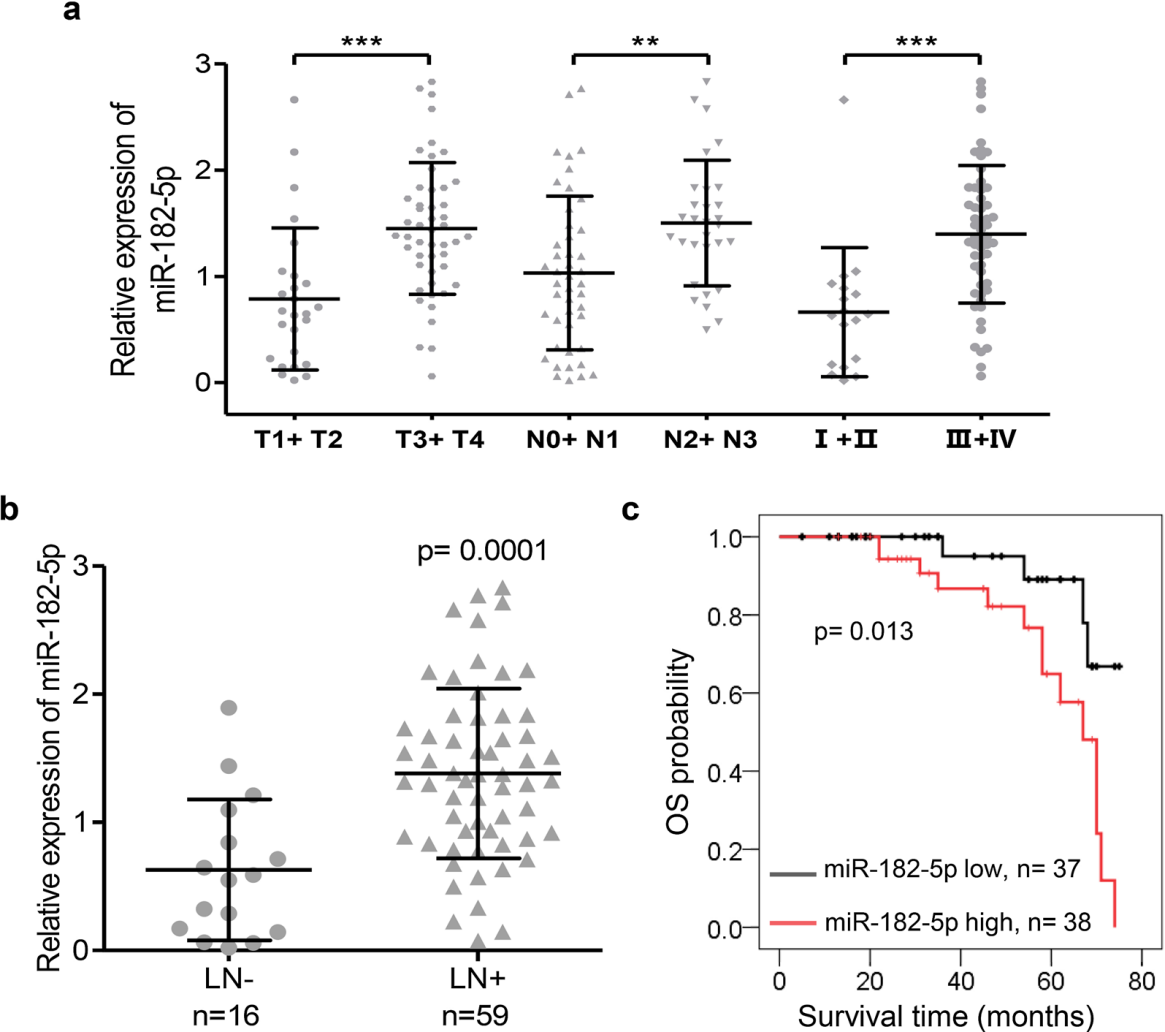
The clinical significance of miR-182-5p in patients with NPC. **a** The relative expression of miR-182-5p in 75 NPC tissues was measured by qRT-PCR. Dot plots present a comparison of miR-182-5p expression in NPC samples stratified by T classification (T1 + T2 vs. T3 + T4), N classification (N0 + N1 vs. N2 + N3), and Clinical Stage (I + II vs. III + IV). Data represent the mean ± SD. **p < 0.01, ***p < 0.001, Mann–Whitney test. **b** qRT-PCR for miR-182-5p was performed in the primary NPC samples without (LN−) or with (LN+) lymph node metastasis. p = 0.0070, Mann–Whitney test. **c** Actuarial probabilities were calculated using the Kaplan–Meier method and compared using the log-rank test (p = 0.013). Patients with high miR-182-5p expression had a significantly worse overall survival (OS) than those with low miR-182-5p expression

To evaluate the clinical significance of miR-182-5p in human NPC, the expression level of miR-182-5p was analyzed in seventy-five NPC samples using qRT-PCR. The relationship between miR-182-5p expression status and clinicopathological features of NPC was evaluated. miR-182-5p overexpression was significantly associated with advanced T classification, poor N classification, high clinical stage (Fig. 1a and Additional file 1: Table S1). No significant differences were found in the expression of miR-182-5p in regards to age and gender (Additional file 1: Table S1). In addition, miR-182-5p up-regulation was closely associated with lymph node metastasis (p = 0.0070) (Fig. 1b). Moreover, Kaplan–Meier analysis indicated that high miR-182-5p expression was closely associated with NPC poor overall survival (Fig. 1c). These results indicated that miR-182-5p overexpression might play an important role in the regulation of NPC progression.

### miR-182-5p promotes NPC cell proliferation and migration in vitro and tumorigenesis and tumor metastasis in vivo

**Fig. 2:**
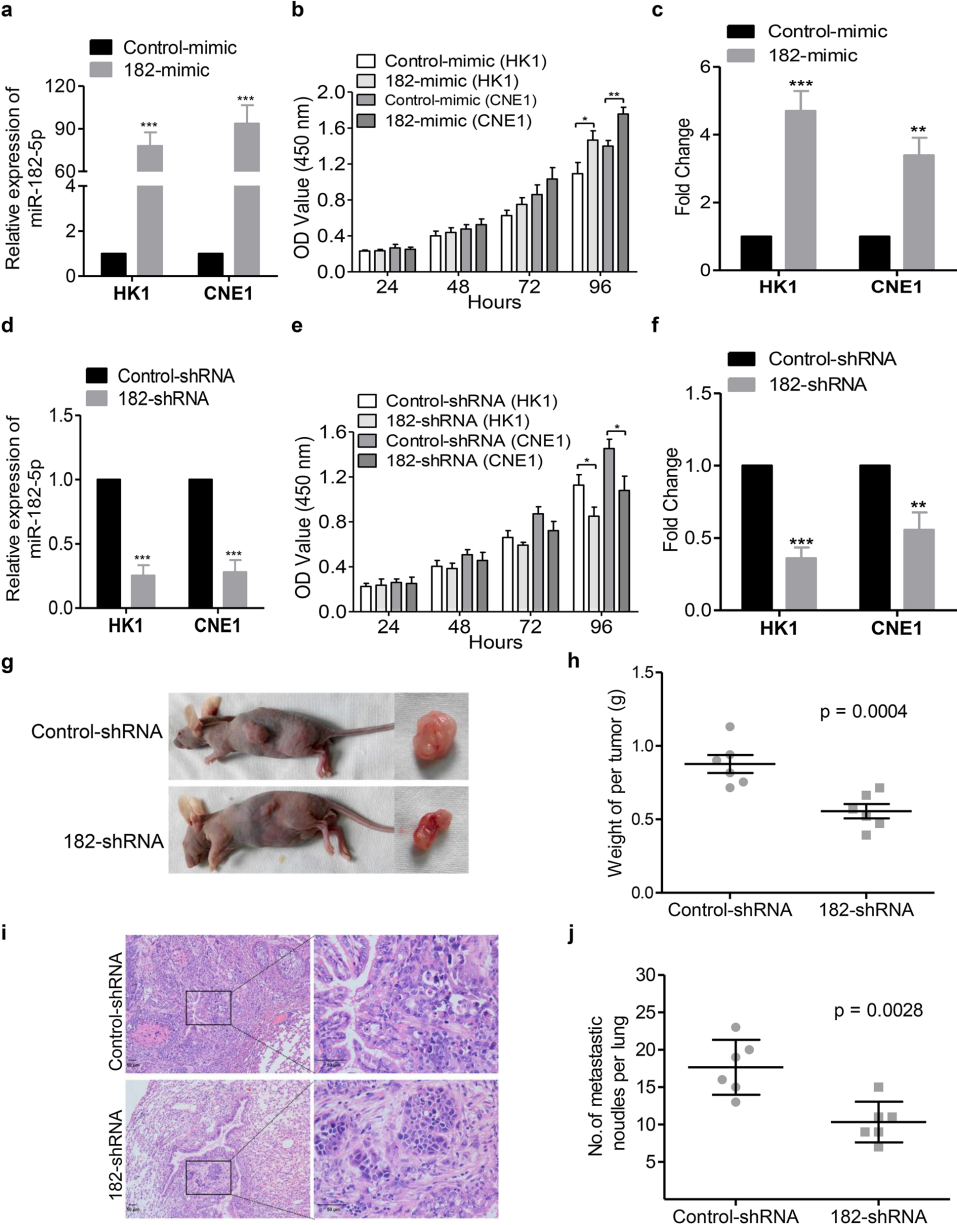
Functional study of miR-182-5p. **a**, **d** The relative expression of miR-182-5p was validated by qRT-PCR in cell lines transfected with the indicated oligonucleotides/plasmids. **b**, **e** Cell growth of the indicated cell lines in vitro was measured at different time points as indicated by CCK-8 assay. **c**, **f** The migratory abilities of the indicated cell lines were measured by transwell cell migration assay. Re-introduction of miR-182-5p promoted (**c**), whereas reduction of miR-182-5p inhibited (**f**) NPC cell migration. **g**, **h** HK1 cells stably infected with lentivirus containing Control-shRNA or miR-182-5p shRNA (182-shRNA) was inoculated subcutaneously into nude mice. The images (**g**) and weight (**h**) of xenograft tumors are shown. p = 0.0004, n = 6. **i**, **j** The effects of miR-182-5p on tumor metastasis were evaluated by the tail vein-lung metastasis model. **i** H&E staining of pulmonary sections from the tumor-bearing mice (scale bars = 50 μm). **j** Numbers of metastatic nodules per lung in the nude mice were summarized. p = 0.0189, n = 6. All data represent the mean ± SD from three independent experiments. *p < 0.05, **p < 0.01 and ***p < 0.001, Student’s *t*-test

In order to explore the biological function of miR-182-5p in NPC, miR-182-5p mimic was transfected into HK1 and CNE1 cells by using Lipofectamine 2000, and Control-mimic microRNA transfected cells were used as controls (Fig. 2a). Then, the effects of miR-182-5p on cell growth and migration were evaluated. CCK-8 assays showed that miR-182-5p overexpression increased the cell growth (Fig. 2b). Besides, cell migration assay revealed that miR-182-5p mimic could dramatically increase cell motility compared with the Control-mimic treated cells (Fig. 2c and Additional file 1: Fig. S1). In contrast, suppression of miR-182-5p by using the microRNA shRNA lentivirus impeded cell growth and migration in both NPC cell lines (Fig. 2d–f and Additional file 1: Fig. S1).

To further determine the function of miR-182-5p in the growth of NPC in vivo, we performed NPC xenograft growth in the nude mice. HK1 cells stably infected with lentivirus containing miR-182-5p shRNA were inoculated subcutaneously into double dorsal flanks of 4-week-old nude mice. As shown in Fig. 2g, h, the growth of tumors derived from HK1 cells stably infected with the miR-182-5p shRNA were significantly suppressed compared with that of xenografts derived from cells infected with control negative microRNA shRNA. Besides, knockdown of miR-182-5p induced a significant decrease of the numbers of lung metastatic lesions compared with the group treated with Control-shRNA (Fig. 2i, j). Therefore, miR-182-5p might positively regulate tumorigenesis and metastasis in NPC.

### miR-182-5p inhibits ZFP36L1 expression by targeting its 3′ UTR in NPC cells

**Fig. 3:**
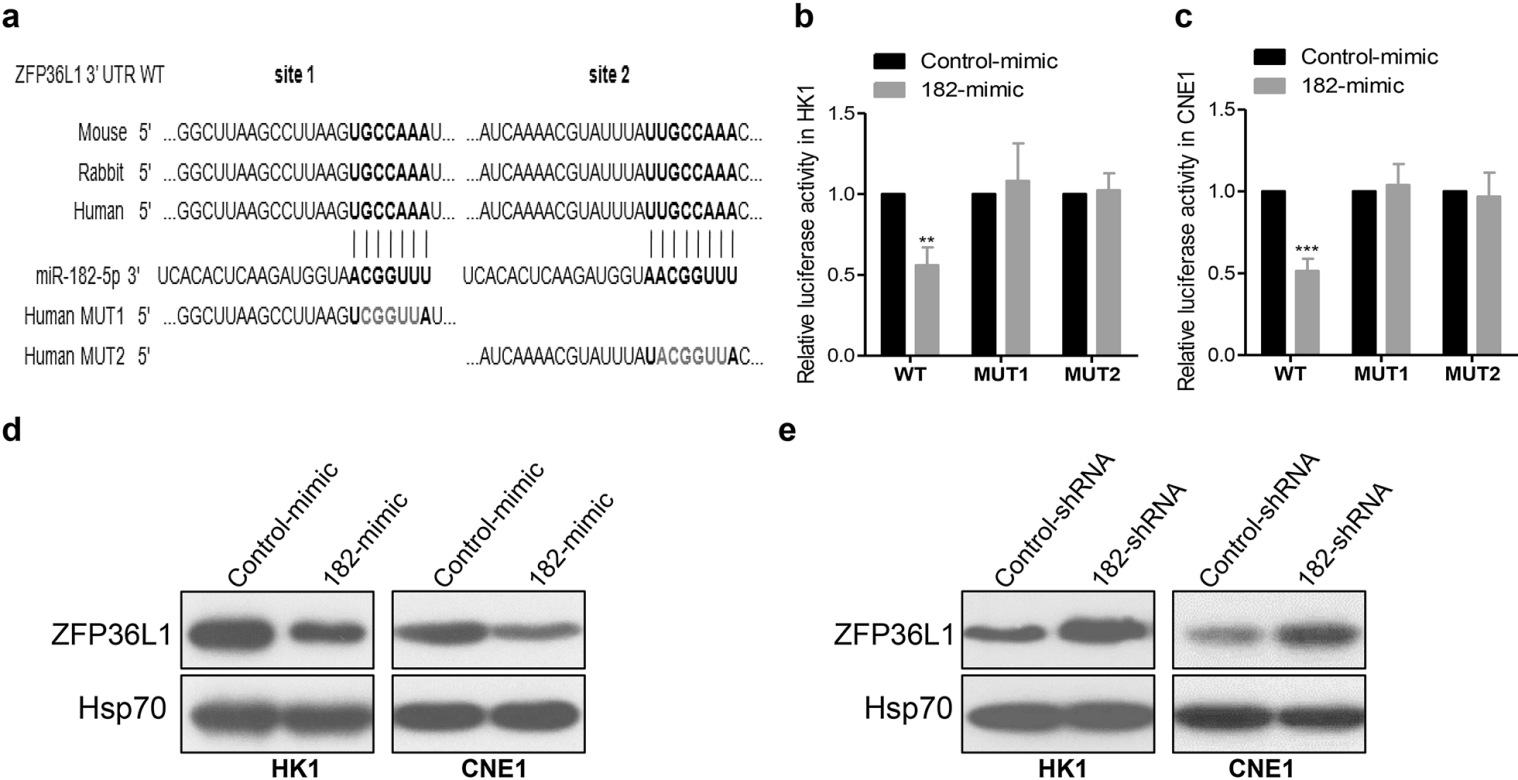
miR-182-5p inhibited ZFP36L1 expression by targeting its 3′ UTR in NPC cells. **a** The predicted duplex formation between the 3′ UTR of ZFP36L1 and miR-182-5p was shown. The wild-type (WT) and two mutants (MUT1 and MUT2) binding sites of the ZFP36L1 3′ UTR to miR-182-5p were cloned into the psiCHECK-2 vector. **b**, **c** Luciferase reporter assays were performed in HK1 (**b**) and CNE1 (**c**) cells co-transfected with Control mimic or miR-182-5p mimic together with the luciferase vectors containing the wide-type or mutant ZFP36L1 3′ UTR. **p < 0.01 and ***p < 0.001, Student’s *t*-test. **d**, **e** The expression of the indicated proteins was determined by Western blot analysis. ZFP36L1 was down-regulated by miR-182-5p mimic (**d**), but up-regulated by miR-182-5p inhibitor (**e**)

To identify the potential targets of miR-182-5p, we integrated bioinformatic algorithms using the publicly available databases miRanda (http://www.microrna.org), miRDB (http://www.mirdb.org), TargetScan (http://www.targetscan.org). According to the bioinformatics analysis, two putative binding sites for miR-182-5p was found at the 3′ UTR of ZFP36L1 (also known as TIS11B, BRF1, Berg36, ERF-1) (Fig. 3a). Then, we performed luciferase reporter assays using the 3′ UTR of ZFP36L1. As shown in Fig. 3b, c, decreased luciferase activity of the wild-type ZFP36L1 3′ UTR in miR-182-5p overexpressed cells was observed. Besides, mutation in potential interaction sites 1 or 2 abrogated the miR-182-5p-induced effect on the luciferase activity of ZFP36L1 3′ UTR (Fig. 3b, c). Moreover, the protein levels of ZFP36L1 were down-regulated in cells transfected with the miR-182-5p mimic (Fig. 3d), whereas knockdown of miR-182-5p resulted in up-regulation of the ZFP36L1 protein (Fig. 3e). All these data demonstrated that ZFP36L1 was a potential functional target of miR-182-5p in NPC.

### ZFP36L1 may function as a tumor-suppressor in NPC and its expression levels are inversely correlated with that of miR-182-5p

Previously, Hodson’s group have established a role for ZFP36L1 in the prevention of malignant transformation [19]. To determine the function of ZFP36L1 in NPC, we generated cell lines expressing ectopic ZFP36L1 in HK1 and CNE1 cells (Additional file 1: Fig. S2a). On the other hand, siRNA specifically targeting ZFP36L1 were also used in these cells (Additional file 1: Fig. S2a). Overexpression of ZFP36L1 suppressed the cell growth and migration compared with the controls (Additional file 1: Fig. S2b, d). Conversely, ZFP36L1 knockdown enhanced cell proliferation and migration in both HK1 and CNE1 cells (Additional file 1: Fig. S2c, e).

**Fig. 4:**
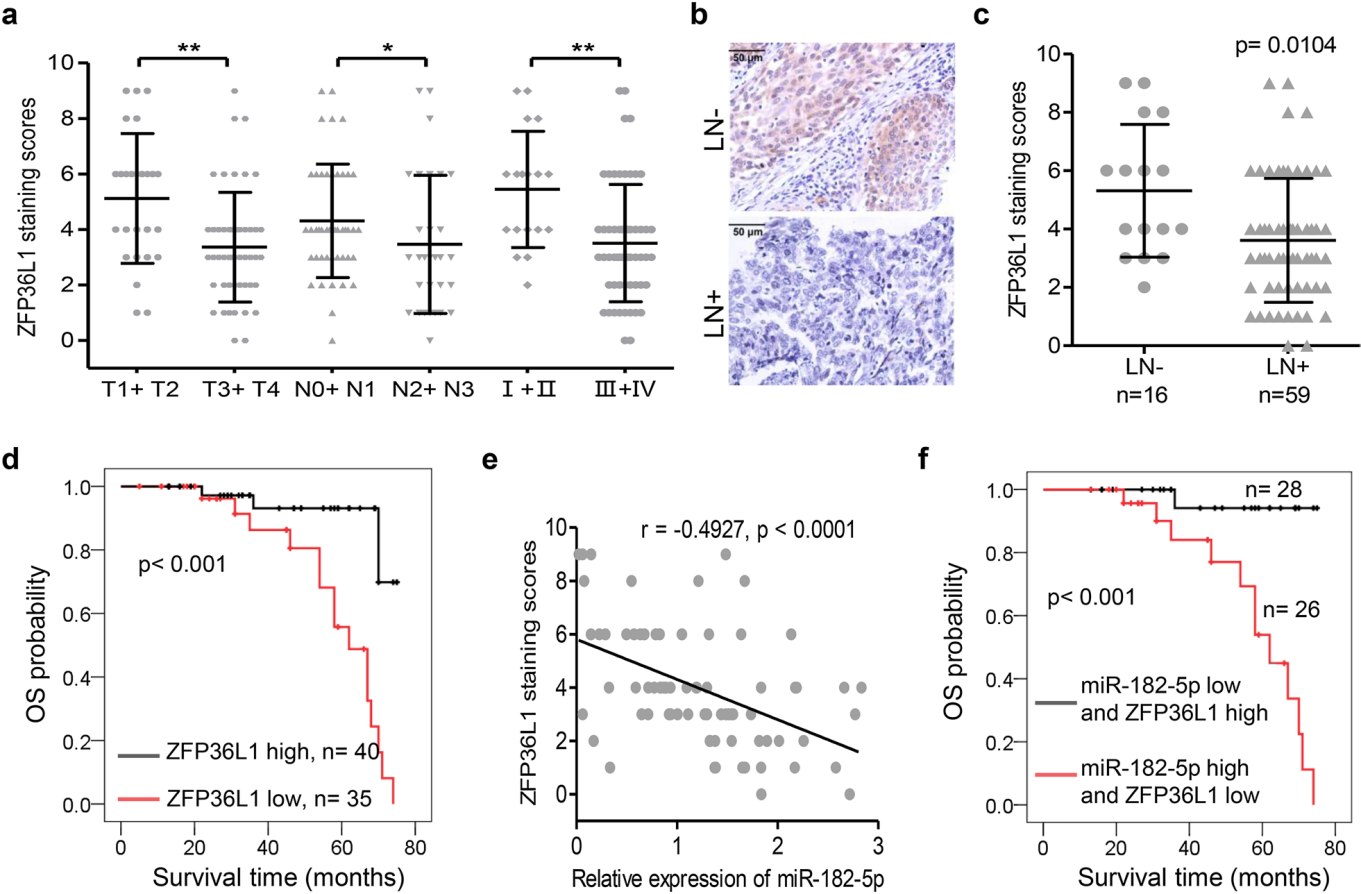
The clinical significance of ZFP36L1 in NPC patients and an inverse correlation was observed between miR-182-5p and ZFP36L1 in cancer tissues. **a** IHC for ZFP36L1 was performed in seventy-five clinical NPC samples. Dot plots present a comparison of ZFP36L1 expression in NPC tissues stratified by T classification (T1 + T2 vs. T3 + T4), N classification (N0 + N1 vs. N2 + N3), and Clinical Stage (I + II vs. III + IV). Data represent the mean ± SD. *p < 0.05, **p < 0.01, Mann–Whitney test. **b**, **c** IHC was performed in the primary NPC samples without (LN−) or with (LN+) lymph node metastasis. The panel **b** presents representative images, while the panel **c** illustrates the statistical results using a Mann–Whitney test. Scale bars, 50 μm. **d** Overall survival curves were generated based on the ZFP36L1 protein expression status in tumor tissues. Actuarial probabilities were calculated using the Kaplan–Meier method and compared using the log-rank test. **e** There was a negative correlation of ZFP36L1 staining scores and miR-182-5p relative expression levels in NPC tissue samples (n = 75; r = − 0.4927, p < 0.0001; linear regression analysis). **f** Up-regulation of miR-182-5p and down-expression of ZFP36L1 (**e**) were closely associated with poor overall survival rates of NPC patients

To further measure the relationship between ZFP36L1 and miR-182-5p in NPC, the ZFP36L1 protein expression in each primary NPC sample was evaluated using IHC. As shown in Fig. 4a and Additional file 1: Table S2, ZFP36L1 down-regulation was closely associated with advanced tumor invasion (p = 0.013), advanced lymph node metastasis (p = 0.018), advanced clinical stage (p = 0.001). Notably, the expression of ZFP36L1 in NPC patient with lymph node metastasis was significantly down-regulated (Fig. 4b, c). Patients with low ZFP36L1 expression showed shortening overall survival when compared to that with high ZFP36L1 expression (p < 0.001, Fig. 4d). Additionally, the expression levels of miR-182-5p and ZFP36L1 were inversely correlated in NPC tissues (Fig. 4e). Furthermore, the combination of low miR-182-5p expression and high ZFP36L1 expression was more significantly correlated with the overall survival time of patients with NPC (Fig. 4f). Taken together, These results suggested that the miR-182-5p/ZFP36L1 axis was a crucial factor affecting NPC patients’ clinical outcomes.

### miR-182-5p enhances NPC cell proliferation and migration depends on the down-regulation of ZFP36L1

**Fig. 5:**
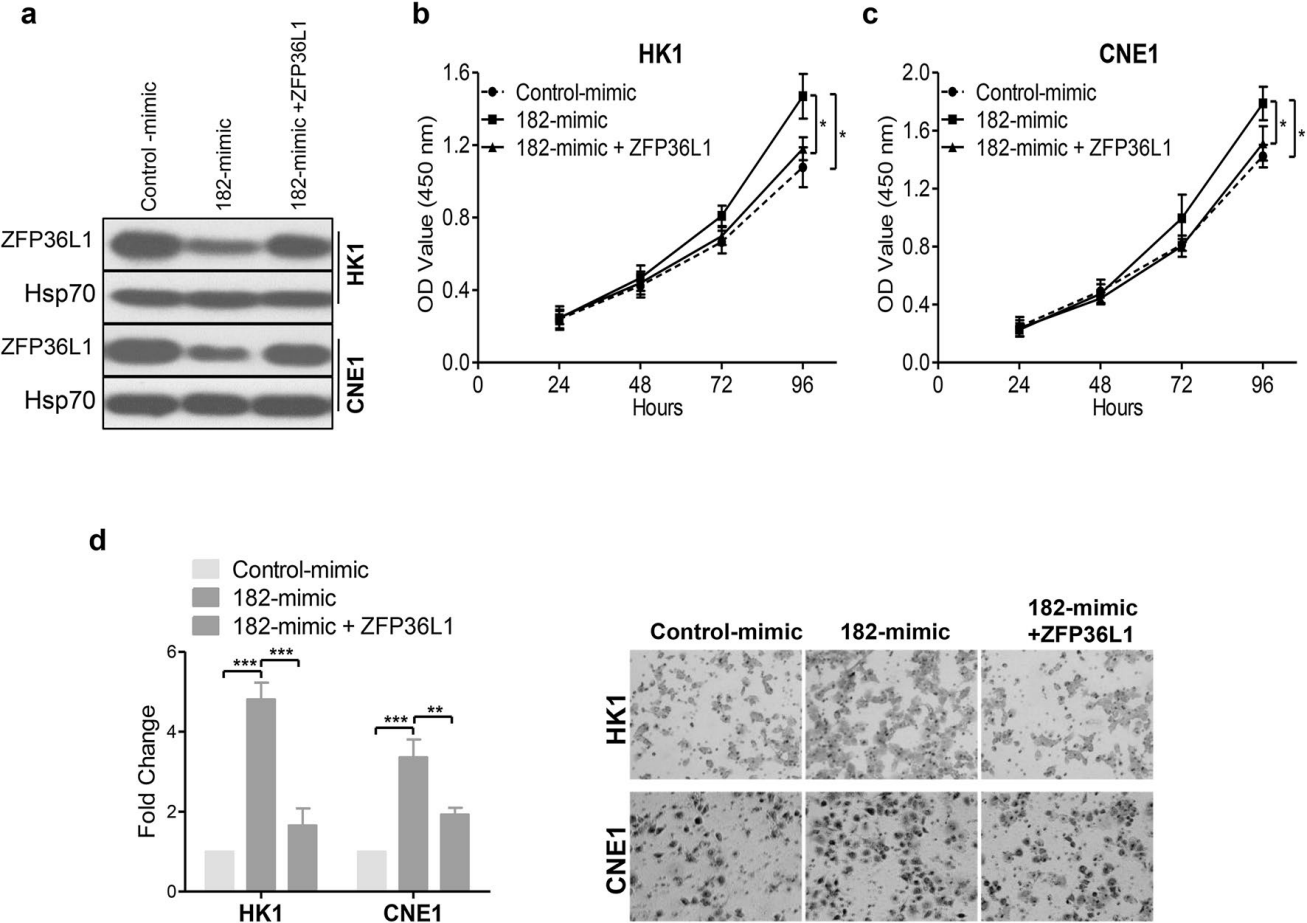
miR-182-5p promoted cell proliferation and migration by targeting ZFP36L1 in NPC. **a** ZFP36L1 expression was examined by Western blot analysis in cells transfected with miR-182-5p and/or ZFP36L1-expressing vector. **b**, **c** CCK-8 assays were performed in HK1 (**b**) and CNE1 (**c**) cells treated as described in **a**. **d** The promotion effect of miR-182-5p on NPC cell migration depends on the down-regulation of ZFP36L1. Cell migration assays were performed in the indicated cells (×200). **p < 0.01 and ***p < 0.001, Student’s *t*-test

Next, we sought to determine whether miR-182-5p promoted NPC cell proliferation and migration by down-regulating ZFP36L1. ZFP36L1 was transiently transfected into HK1 and CNE1 cells expressing miR-182-5p mimic (Fig. 5a). Results of CCK-8 assays showed that re-introduction of ZFP36L1 reversed the increase in cell growth induced by miR-182-5p overexpression (Fig. 5b, c). Moreover, the migratory ability was almost rescued to the original levels in miR-182-5p-transfected cells with ectopic overexpressing ZFP36L1 (Fig. 5d). Collectively, these results indicated that miR-182-5p-mediated promotion of cell proliferation and migration depends on the down-regulation of ZFP36L1 in NPC.

### HIF-1α is involved in the up-regulation of miR-182-5p in NPC cells

**Fig. 6:**
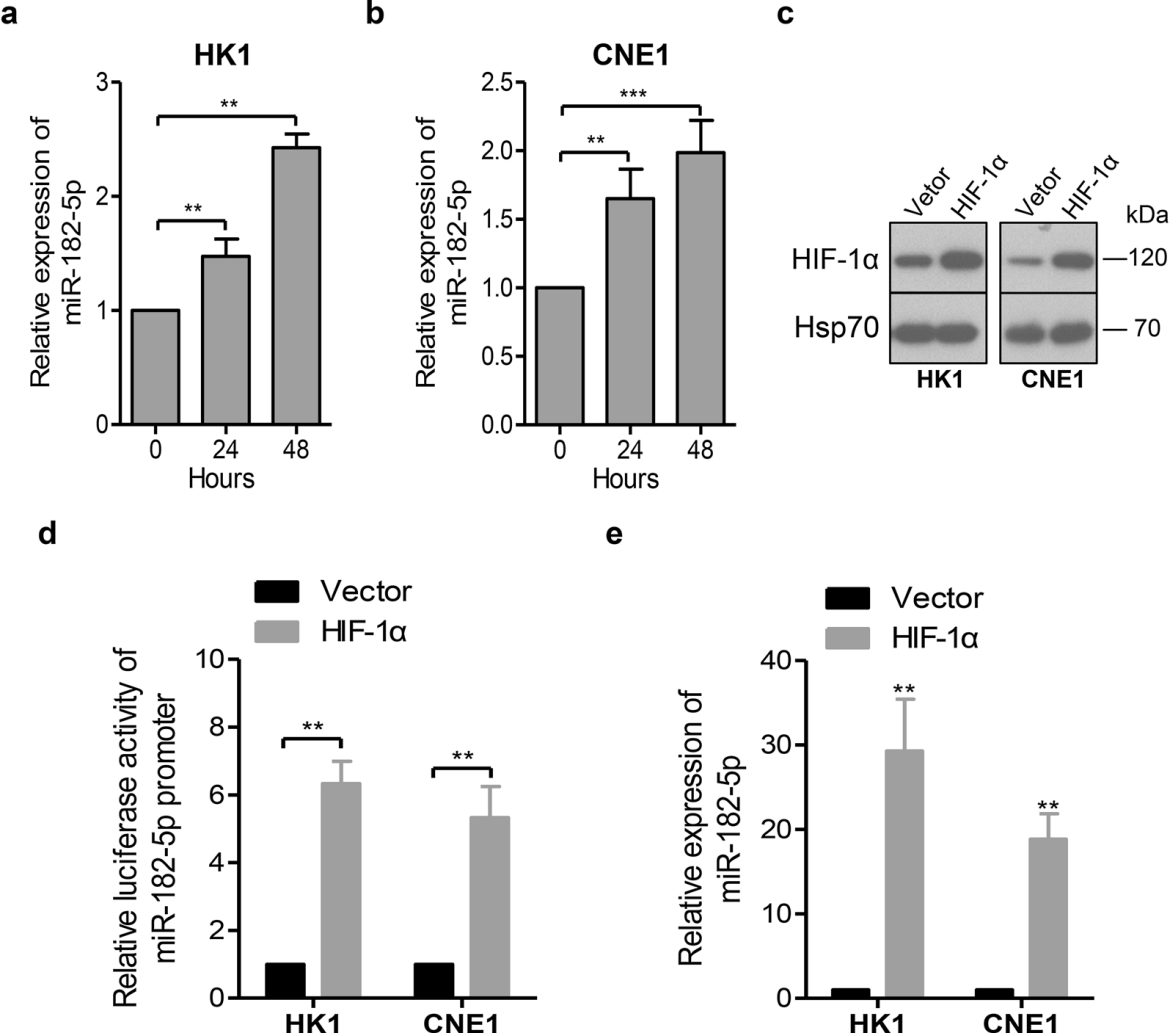
HIF-1α was involved in the up-regulation of miR-182-5p in NPC cells. **a**, **b** HK1 and CNE1 cells were exposed to 1% oxygen tension for 0 h, 24 or 48 h, and miR-182-5p levels were measured by qPCR. **c** Validation of cells expressing the indicated plasmids. HK1 and CNE1 cells overexpressing Vector or HIF-1α, as indicated, were subjected to western blot analysis. **d** The luciferase reporters containing a miR-182-5p promoter region was co-transfected with control Vector or HIF-1α into HK1 and CNE1 cells. Luciferase activities were detected after 48 h of transfection. **e** The relative expression of miR-182-5p was measured by qRT-PCR in cell lines overexpressing Vector or HIF-1α, as indicated. All data represent the mean ± SD from three independent experiments. *p < 0.05, **p < 0.01 and ***p < 0.001, Student’s *t*-test

Given that hypoxia is associated with increased malignancy, resistance to therapy and distant metastasis in solid tumors, including NPC [20, 21], we hypothesized that hypoxia may mediate the up-regulation of miR-182-5p in NPC. To this end, miR-182-5p levels were measured by qPCR in cells cultured in the hypoxic incubator at different time points. Interestingly, miR-182-5p was significantly induced in a time-dependent manner in response to hypoxia (Fig. 6a, b). As the cellular adaptive responses to hypoxic stress are mainly mediated by the hypoxia-inducible factors (HIFs), of which HIF-1α is the best-examined subunit binding to hypoxia-responsive element (HRE) sites in the promoters of target genes [22, 23], we tested whether miR-182-5p was transcriptionally induced by the HIF-1α. Indeed, by searching the ~ 2400 bp promoter sequence of miR-182-5p, three potential HRE sites were identified (Additional file 1: Fig. S3). As illustrated in Fig. 6c, d, ectopic overexpression of HIF-1α significantly increased miR-182-5p promoter activity. Consistently, the expression levels of miR-182-5p were increased by HIF-1α overexpression (Fig. 6e). These data suggested that miR-182-5p overexpression in NPC was partly due to the transcriptional activation effect induced by HIF-1α.

## Discussion

**Fig. 7:**
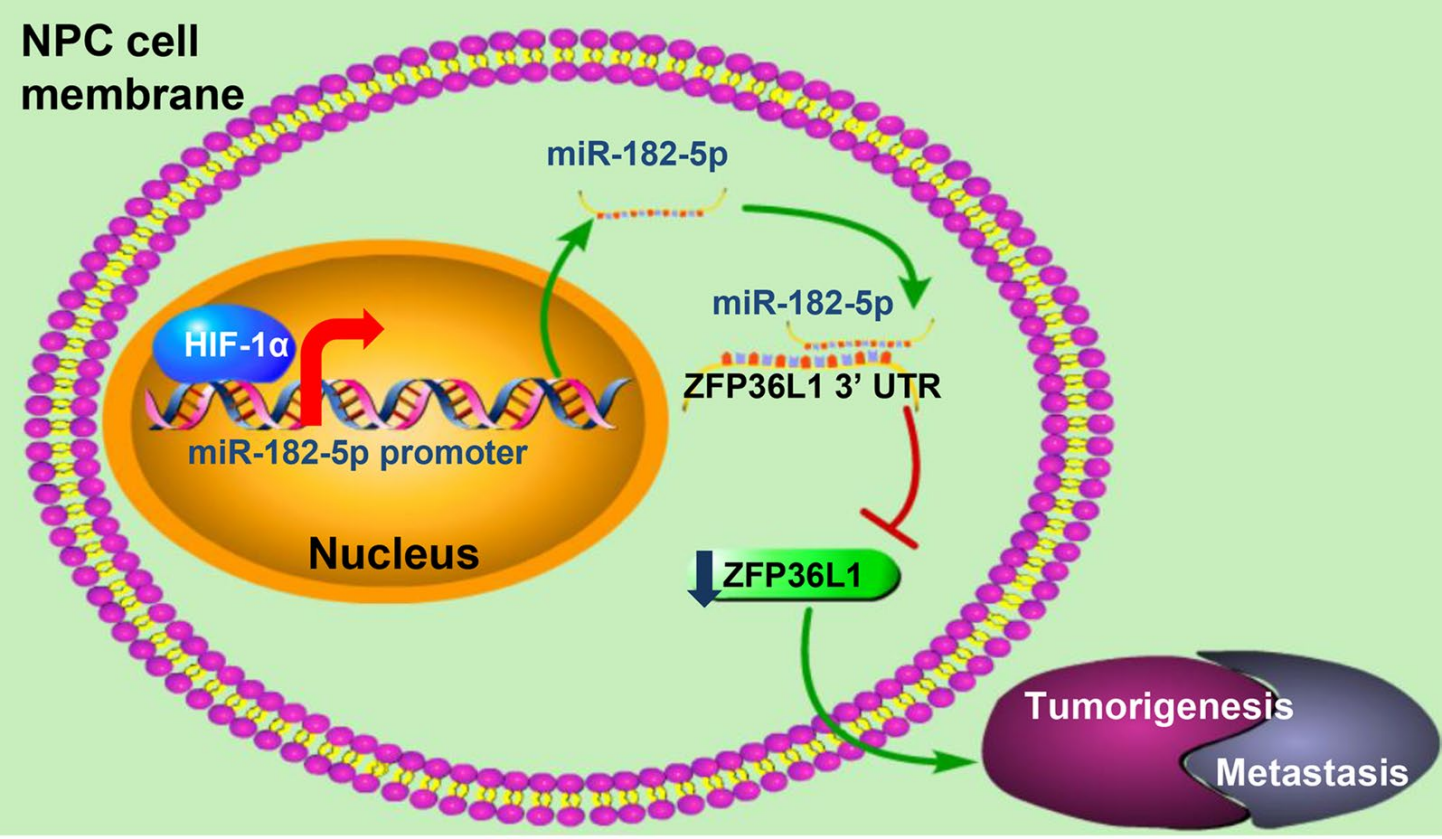
The proposed model for the HIF-1α/miR-182-5p/ZFP36L1 signaling in NPC tumorigenesis and metastasis. Overexpression of miR-182-5p, the direct target of HIF-1α, results in down-regulation of ZFP36L1 and consequently promotes NPC progression

In the current study, as shown in Fig. 7, we have provided evidence that miR-182-5p may function as an oncogenic role in NPC, by promoting tumorigenesis and tumor metastasis. HIF-1α is recruited to miR-182-5p promoter to transcriptionally upregulate miR-182-5p, which facilitates NPC progression by targeting the tumor suppressor ZFP36L1.

Over the past twenty years, more and more efforts have been made to disclose the association of miRs with cancer by acting as oncogenes or tumor suppressors [24, 25]. In regards to miR-182-5p, there is increasing evidence that it plays an oncogenic role in many cancers. For example, miR-182 is elevated and positively regulates tumor development and progression in breast cancer [12, 26], suggesting that miR-182 might be an attractive therapeutic target for patients with breast cancer. In contrast, the tumor suppressor role of miR-182 in gastric cancer has been documented [27]. However, the potential functions and mechanisms of miR-182-5p in NPC remains poorly understood. In this study, we confirmed that miR-182-5p promoted NPC cell proliferation, migration, tumorigenesis and metastasis. Moreover, miR-182-5p overexpression was associated with NPC development and poor prognosis.

As a member of the Tristetraprolin (TTP) RNA binding protein family, ZFP36L1 regulate gene expression post-transcriptionally by promoting mRNA decay [28, 29]. In mammals, ZFP36L1 and its family members have been shown to regulate development [30], cell differentiation [31], cell cycle [32], apoptosis [33] and anti-cancer capability [19, 34] by targeting an extensive overlapping repertoire of mRNAs. In the present study, we have demonstrated that ZFP36L1 was a functional target of miR-182-5p in NPC. Functional studies established that ZFP36L1 repressed NPC cell proliferation and migration ability. Clinical analyses showed that ZFP36L1 expression levels were inversely correlated with the levels of miR-182-5p. Moreover, ZFP36L1 down-regulation was associated with NPC poor prognosis, suggesting the tumor-suppressor role of ZFP36L1 in NPC.

Hypoxia, or low oxygen tension, is implicated in a wide range of biological and cellular processes, such as cell survival, cell proliferation and migration [20, 35]. The primary mediator that controls those processes is HIF-1α, which has been identified as the best characterized oxygen sensor and regulator of the hypoxic-adaptive responses [36]. It has been previously shown that the expression of miR-210, a demonstrated master hypoxamir, is regulated by HIF-1α in a variety of tumor types [37]. Here, miR-182-5p was confirmed to be hypoxia-induced in human NPC cells. To determine whether HIFs contributes to the hypoxic induction of miR-182-5p, we performed the luciferase assays and demonstrated the transcriptional activation of miR-182-5p by HIF-1α.

## Conclusions

In summary, our research has revealed that the up-regulation of miR-182-5p in NPC is attributed to, at least in part, the HIF-1α-dependent transcriptional activation. miR-182-5p plays a crucial role in promoting NPC tumorigenesis and metastasis by silencing ZFP36L1, a molecule that appears to be an attractive prognostic factor for NPC. This novel HIF-1α/miR-182-5p/ZFP36L1 pathway may be valuable to design new treatments for the patients with NPC.

## Supplementary Information

**Additional file 1: Figure S1.** miR-182-5p promoted cell migration in NPC. Representative images of cell migration assays performed in the indicated cells were shown (×200). These experiments were repeated at least three times. **Figure S2.** ZFP36L1 suppressed cell proliferation and migration in NPC. **a** Re-expression or knockdown of ZFP36L1in the indicated cells was confirmed by Western blot analysis. **b** ZFP36L1 overexpression significantly reduced the cell viabilities. Cells were transfected with Vector or ZFP36L1 for 96 h. The cell growth rates were determined by CCK-8 assays. **c** Knockdown of ZFP36L1 remarkably increased the cell survival. Cells were treated with siControl or siZFP36L1 for 96 h. **d**, **e** The migratory abilities of the indicated cells were measured by transwell cell migration assay. All data represent the mean ± SD from three independent experiments. *p < 0.05, **p < 0.01 and ***p < 0.001, Student’s *t*-test. **Figure S3. **The ~ 2400 bp promoter sequence of miR-182-5p was showed and three potential HRE sites were identified according to the consensus sequence (A/G)CGTG. TSS, transcriptional start site. **Table S1.** Correlation of miR-182-5pexpression and clinical features of patients with NPC. **Table S2.** Correlation of ZFP36L1 expression and clinical features of patients with NPC.

## Data Availability

All data generated or analyzed during this study are included in this published article. Further details are available from the corresponding author upon request.

## Abbreviations

NPC: Nasopharyngeal carcinoma
miR-182-5p: microRNA 182-5p
ZFP36L1: ZFP36 ring finger protein like 1
3′ UTR: 3′ Un-translated region
HIF-1α: Hypoxia-inducible factor 1α
HRE: Hypoxia-responsive element
TTP: Tristetraprolin
qRT-PCR: Quantitative real-time polymerase chain reaction
IHC: Immunohistochemistry
