# Supplementary material for: The oncogenic role of HIF-1α/miR-182-5p/ZFP36L1 signaling pathway in nasopharyngeal carcinoma
Source: Cancer Cell Int. 2021 Aug 31;21:462. doi: 10.1186/s12935-021-02177-3 (PMC8406720; doi:10.1186/s12935-021-02177-3)
Supplement: Supplementary file 1 — Additional file 1: Figure S1. miR-182-5p promoted cell migration in NPC. Representative images of cell migration assays performed in the indicated cells were shown (×200). These experiments were repeated at least three times. Figure S2. ZFP36L1 suppressed cell proliferation and migration in NPC. a Re-expression or knockdown of ZFP36L1in the indicated cells was confirmed by Western blot analysis. b ZFP36L1 overexpression significantly reduced the cell viabilities. Cells were transfected with Vector or ZFP36L1 for 96 h. The cell growth rates were determined by CCK-8 assays. c Knockdown of ZFP36L1 remarkably increased the cell survival. Cells were treated with siControl or siZFP36L1 for 96 h. d, e The migratory abilities of the indicated cells were measured by transwell cell migration assay. All data represent the mean ± SD from three independent experiments. *p < 0.05, **p < 0.01 and ***p < 0.001, Student’s t-test. Figure S3. The ~ 2400 bp promoter sequence of miR-182-5p was showed and three potential HRE sites were identified according to the consensus sequence (A/G)CGTG. TSS, transcriptional start site. Table S1. Correlation of miR-182-5pexpression and clinical features of patients with NPC. Table S2. Correlation of ZFP36L1 expression and clinical features of patients with NPC. [file 12935_2021_2177_MOESM1_ESM.doc]

Additional file 1


Fig. S1 miR-182-5p promoted cell migration in NPC. Representative images of cell migration assays performed in the indicated cells were shown (200 ×). These experiments were repeated at least three times.

Fig. S2 ZFP36L1 suppressed cell proliferation and migration in NPC. a Re-expression or knockdown of ZFP36L1 in the indicated cells was confirmed by Western blot analysis. b ZFP36L1 overexpression significantly reduced the cell viabilities. Cells were transfected with Vector or ZFP36L1 for 96 hours. The cell growth rates were determined by CCK-8 assays. c Knockdown of ZFP36L1 remarkably increased the cell survival. Cells were treated with siControl or siZFP36L1 for 96 hours. d, e The migratory abilities of the indicated cells were measured by transwell cell migration assay. All data represent the mean ± SD from three independent experiments. *p < 0.05, **p < 0.01 and ***p < 0.001, Student's t-test. 

Fig. S3 The ~2400bp promoter sequence of miR-182-5p was showed and three potential HRE sites were identified according to the consensus sequence (A/G)CGTG. TSS, transcriptional start site. 

Table S1	Correlation of miR-182-5p expression and clinical features of patients with NPC.
Characteristics	Cases	miR-182-5p expression	p valuea	
		Low (%) 	High (%)		
Age (years old)				0.133	
  <52 	37	15 (40.5)	22 (59.5)		
  ≥52 	38	22 (57.9)	16 (42.1)		
Gender				0.119	
  Male	57	31 (54.4)	26 (45.6)		
  Female	18	6 (33.3)	12 (66.7)		
T classification				0.000	
  T1+ T2	26	21(80.8)	5 (19.2)		
  T3+ T4	49	16 (32.7)	33 (67.3)		
N classification				0.024	
  N0+ N1	45	27 (60.0)	18 (40.0)		
  N2+ N3	30	10 (33.3)	20 (66.7)		
Death				0.018	
  No	57	33 (57.9)	24 (42.1)		
  Yes	18	4 (22.2)	14 (77.8)		
Clinical Stage				0.000	
 Ⅰñ+Ⅱò	18	17 (94.4)	1 (5.6)		
 Ⅲó+Ⅳô	57	20 (35.1)	37 (64.9)		
a Chi-square test. 	

Table S2	Correlation of ZFP36L1 expression and clinical features of patients with NPC. 
Characteristics	Cases	ZFP36L1 expression	p valuea	
		Low (%) 	High (%) 		
Age (years old)				0.422	
  <52 	37	19 (51.4)	18 (48.6)		
  ≥52 	38	16 (42.1)	22 (57.9)		
Gender				0.159	
  Male	57	24 (42.1)	33 (57.9)		
  Female	18	11 (61.1)	7 (38.9)		
T classification				0.013	
  T1+ T2	26	7 (26.9)	19 (73.1)		
  T3+ T4	49	28 (57.1)	21 (42.9)		
N classification				0.018	
  N0+ N1	45	16 (35.6)	29 (64.4)		
  N2+ N3	30	19 (63.3)	11 (36.7)		
Death				0.001	
  No	57	20 (35.1)	37 (64.9)		
  Yes	18	15 (83.3)	3 (16.7)		
Clinical Stage				0.003	
 Ⅰñ+Ⅱò	18	3 (16.7)	15 (83.3)		
 Ⅲó+Ⅳô	57	32 (56.1)	25 (43.9)		
a Chi-square test. 	
